# Additions to *Diatrypaceae* (*Xylariales*): Novel Taxa and New Host Associations

**DOI:** 10.3390/jof9121151

**Published:** 2023-11-28

**Authors:** Naghmeh Afshari, Omid Karimi, Antonio R. Gomes de Farias, Nakarin Suwannarach, Chitrabhanu S. Bhunjun, Xiang-Yu Zeng, Saisamorn Lumyong

**Affiliations:** 1 Department of Biology, Faculty of Science, Chiang Mai University, Chiang Mai 50200, Thailand; naghmeh.afshar20@gmail.com; 2 Center of Excellence in Fungal Research, Mae Fah Luang University, Chiang Rai 57100, Thailand; karimiomid18@gmail.com (O.K.); avnishbhunjun@gmail.com (C.S.B.); 3 Center of Excellence in Microbial Diversity and Sustainable Utilization, Chiang Mai University, Chiang Mai 50200, Thailand; suwan.462@gmail.com; 4 School of Science, Mae Fah Luang University, Chiang Rai 57100, Thailand; 5 Department of Plant Pathology, College of Agriculture, Guizhou University, Guiyang 550025, China; xyzeng3@gzu.edu.cn; 6 Academy of Science, The Royal Society of Thailand, Bangkok 10300, Thailand

**Keywords:** new taxa, diatrypaceous, fungal taxonomy, saprobic fungi, *Sordariomycetes*

## Abstract

*Diatrypaceae* members have a broad distribution and are commonly found on decaying wood. Despite taxonomic and morphological challenges within this group, there has been a growing interest in *Diatrypaceae* in recent years. The dead branches of several plant hosts with fungal fruiting bodies were collected from Doi Tung National Park, Chiang Rai, and the Narathiwat Provinces in Thailand. Their morphological characteristics, coupled with a molecular phylogeny of combined ITS and *tub*2 sequence data, were used to introduce two novel *Allodiatrype* species (*A. dalbergiae* and *A. eleiodoxae*) and one new *Melanostictus* species (*M. chiangraiensis*). Moreover, four new host records, *Diatrypella heveae*, *D. major*, *Melanostictus thailandicus*, and *Paraeutypella citricola* on *Microcos paniculata*, *Nayariophyton zizyphifolium*, *Dalbergia cultrata*, and *M. paniculata*, respectively, as well as a new geographical record of *D. major* are reported. This research provides detailed descriptions of macro- and microcharacteristics, coupled with a phylogenetic tree for the newly introduced species and host records. The morphological features of *Allodiatrype* and *Melanostictus* are listed in the synoptic table.

## 1. Introduction

Members of *Diatrypaceae* have a widespread distribution in aquatic and terrestrial environments [1,2,3,4,5,6,7,8,9] with diverse lifestyles, such as saprobes, endophytes, and pathogens, on a wide range of crops and woody plants [3,4,5,10,11,12,13,14,15]. Most genera in this family are wood-dwelling [6,12,13,15,16,17,18,19,20,21]. Nevertheless, some cause diseases such as dieback, cankers, and grapevine trunk in *Cryptosphaeria populina*, *C. pullmanensis*, *Cryptovalsa rabenhorstii*, *Eutypa leptoplaca*, *E. lata*, *E. consobrina*, and *E. parasitica* [22,23,24,25,26]. Members of this group produce extracellular ligninolytic enzymes that degrade plant cell walls, consequently facilitating the process of wood decomposition [27,28].

Nitschke [29] erected *Diatrypaceae* as a member of *Xylariales* Nannf. (in *Sordariomycetes*), with *Diatrype* Fr. as the type genus, to accommodate *Calosphaeria* Tul. and C. Tul., *Diatrype* Fr., *Diatrypella* (Ces. & De Not.) De Not., *Quaternaria* Tul. and C. Tul., and *Scoptria* Nitschke [29]. Based on its phylogeny, estimation of divergence time, and evolution of major lineages in the *Xylariales*, *Diatrypaceae* is well-supported in this order, and its divergence has affinities with many families in the *Xylariales* at 66–252 million years ago [30,31]. Recently, several new species have been introduced in this family, and currently, *Diatrypaceae* comprises 27 genera [12,13,15,20,21,32,33,34,35].

Previously, *Diatrypaceae* were primarily classified according to stromatal characteristics, comprising the stromatal development degree, perithecial neck structure, and host tissue type [36,37]. However, this family is quite perplexed by the morphology of the stomata [38]. In general, members of *Diatrypaceae* have been characterized by eustromatic or pseudostromatic stromata ranging from erumpent to immersed and scarcely superficial. The stromata are mostly black or dark brown. The perithecial ascomata have ostiolar necks, and the asci are eight-spored or polysporous, with some being scarcely one or two-spored and unitunicate. The ascospores are ellipsoidal, globose, filiform, or allantoid, and they are hyaline–light brown in the sexual morph. In the asexual morph, they are acervulus subcortical, erumpent conidiomata, and hyaline, filiform, curved, or rarely straight conidia [23,39,40,41].

The placement of genera in this family is confusing because many are polyphyletic [6]. Moreover, diatrypaceous taxa are difficult to distinguish based only on morphology, as they share similar morphological characters [1,2]. Therefore, a polyphasic approach must be applied based on at least the morphological features and multilocus phylogeny for the identification and classification of *Diatrypaceae* taxa [15,21,26,28,42,43].

This research aimed to explore and document unidentified species within the *Diatrypaceae* family in the protected area from specific woody plants. Additionally, this study contributes to phylogeny, morphology, host preference, and biodiversity studies and, more importantly, expands our knowledge of the diversity in this family.

During our investigation of wood-inhabiting microfungi in terrestrial habitats in Northern Thailand and a peat swamp forest in Southern Thailand, we collected nine isolates of *Diatrypaceae*. Based on morphological comparisons and combined gene phylogenetic analyses of internal transcribed spacer (ITS) and *β*-tubulin (*tub*2), the novel species *A. dalbergiae, A. eleiodoxae*, and *Melanostictus chiangraiensis* were identified, as well as four new host records of *Diatrypella heveae*, *D. major*, *Melanostictus thailandicus*, *Paraeutypella citricola*, on the decaying wood of *Microcos paniculata*, *Nayariophyton zizyphifolium*, *Dalbergia cultrata*, and *M. paniculata*. Additionally, a new geographical record of *D. major* is introduced. Furthermore, the morphological characteristics of *Allodiatrype* and *Melanostictus* members are provided in synoptic tables.

## 2. Materials and Methods

### 2.1. Sample Collection, Fungi Isolation, and Morphological Studies

Fresh samples were collected from the dead branches of particular host plants during wet and dry seasons, with a temperature range of 25–33 °C in Doi Tung National Park, Chiang Rai, and Narathiwat Provinces, Thailand. The deadwood from each host was sorted into separate bags and taken to the laboratory for examination. The macro-morphological characters were assayed and photographed using a camera mounted on an Olympus SZX61 stereo microscope (Olympus Corporation, Tokyo, Japan). The micro-morphological characteristics were obtained using a Nikon ECLIPSE Ni compound microscope (Nikon, Tokyo, Japan) and a fixed Canon 600 D digital camera (Nikon, Tokyo, Japan). The microfungal structures were measured using Tarosoft (R) Image Frame Work (version 0.9.7). Graphic plates were created using Adobe Photoshop v. CS6. 10.0 software (Adobe Systems, San Jose, CA, USA).

Axenic cultures were obtained from a single spore isolation method on potato dextrose agar (PDA) (39 g/L distilled water, Difco potato dextrose), as described in Senanayake et al. [44]. The plates were incubated at 25–28 °C for 2–4 weeks in the dark. Herbarium materials were deposited in the Mae Fah Luang University Fungarium (MFLU), Chiang Rai, Thailand, and ex-type living cultures were deposited in the Mae Fah Luang University Culture Collection (MFLUCC). In addition, nomenclatural novelties are/will be linked to Index Fungorum (http://www.indexfungorum.org, accessed on 15 August 2023), Facesoffungi (FoF) [45], and the Greater Mekong subregion [46] databases.

### 2.2. DNA Extraction and PCR Amplification 

Fresh mycelia were scraped from colonies onto PDA plates and incubated at 25–28 °C for about one week until hyphae covered the plate. The genomic DNA was extracted using the PureDirex Genomic DNA Isolation Kit (Bio-Helix Co. Ltd., Keelung City, Taiwan) following the manufacturer’s protocol. The polymerase chain reactions (PCR) were performed using the primers and conditions summarized in Table 1. The total volume of 25 μL including 12.5 μL 10× PCR Master Mix with dye, 1 μL of 20 picomolar forward and reverse primer, 9.5 μL double-distilled water, and 1 μL (50–500 ng) DNA template. All PCR amplification products were visualized on 1.5% agarose electrophoresis gel stained with DL5000 DNA Fluorescent Loading Dye (FluoroDye™ Green, 6×, SMOBIO Technology, Inc., Hsinchu, Taiwan), with the D2100 DNA Ladder (ExcelBand™ 100 bp) (SMOBIO Technology, Inc., Hsinchu, Taiwan) used as reference. The sequencing of PCR products was performed by Biogenomed Co., Ltd. (Seoul, Republic of Korea).

### 2.3. Alignments and Phylogenetic Analysis

Ninety-three *Diatrypaceae* reference sequences and the outgroups *Xylaria hypoxylon* (CBS 122620) and *Kretzschmaria deusta* (Hoffm.) P.M.D. Martin (CBS 826.72) were downloaded from NCBI GenBank [50] based on BLASTn search results (https://blast.ncbi.nlm.nih.gov/Blast.cgi; accessed on 1 September 2023), and the literature [15,51] (Table 2). The sequences of ITS and *tub*2 were analyzed individually and in combination. Sequence alignments were performed using the online web server MAFFT v.7 (http://mafft.cbrc.jp/alignment/server/index.html; accessed on 1 September 2023) [52], and alignments were trimmed below the gap threshold (-*gt* 0.25) [53] using trimAl v1.2 (http://trimal.cgenomics.org; accessed on 1 September 2023). Individual gene datasets were concatenated using the Sequence Matrix program v.1.7.8 [54].

The analyses of maximum likelihood (ML) and Bayesian inferences (BI) were performed on XSEDE in the CIPRES Science Gateway portal (https://www.phylo.org/; accessed on 1 September 2023) [55]. The ML tree was conducted with RAxML-HPC v.8 on XSEDE [56] and GTRGAMMA as a substitution model with 1000 bootstrap iterations. The BI tree was performed with MrBayes on XSEDE (3.2.7a) [57]. Following the Akaike Information Criterion (AIC) in jModelTest (2.1.6) [58], TIM2+G and TrN+G nucleotide substitution models were selected as the best-fit models for ITS and *tub*2 datasets. To calculate the Bayesian posterior probabilities (BPP), four simultaneous chains were run for 50 million Markov chain Monte Carlo (MCMC) generations, with trees collected every 1000th generation. The first 25% of sampled trees were discarded as burn-in, and the remaining 7500 trees were used to calculate the posterior probability (PP) of each branch [59]. Tracer (version 1.7) was used to check convergence in MCMC trace files through BI phylogeny [60]. The resulting trees were viewed in FigTree v.1.4.0 [61] and edited in Inkspace v.1.2.2 [62]. All newly generated sequences were deposited in GenBank (Table 2).

**Table 2:** Fungal species, strain voucher, and corresponding GenBank accession numbers of the taxa used in the phylogenetic analyses.

Fungal Species	Strain Vouchers	GenBank Accession Numbers		References
		ITS	*tub*2	
*Allocryptovalsa cryptovalsoidea*	HVFIG02	HQ692573	HQ692524	[27]
*Allocryptovalsa sichuanensis*	**HKAS 107017**	MW240633	MW775592	[13]
*Allodiatrype albelloscutata*	**IFRD9100**	OK257020	_	[63]
*Allodiatrype arengae*	**MFLUCC 15-0713**	MN308411	MN340297	[12]
***Allodiatrype dalbergiae***	**MFLUCC 23-0173**	**OR571759**	**OR771026**	**This study**
***Allodiatrype dalbergiae***	**MFLUCC 23-0174**	**OR571760**	**OR591487**	**This study**
***Allodiatrype dalbergiae***	**MFLUCC 23-0175**	**OR571762**	**OR771025**	**This study**
*Allodiatrype elaeidicola*	**MFLUCC 15-0737a**	MN308415	MN340299	[12]
*Allodiatrype elaeidis*	**MFLUCC 15-0708a**	MN308412	MN340298	[12]
***Allodiatrype eleiodoxae***	**MFLUCC 23-0181**	**OR571761**	**OR591484**	**This study**
*Allodiatrype taiyangheensis*	**IFRDCC2800**	OK257021	OK345036	[63]
*Allodiatrype thailandica*	MFLUCC 15-3662	KU315392	_	[64]
*Allodiatrype trigemina*	**FCATAS842**	MW031919	MW371289	[65]
*Alloeutypa flavovirens*	CBS 272.87	AJ302457	DQ006959	[66]
*Alloeutypa milinensis*	**FCATAS4309**	OP538689	OP557595	[51]
*Anthostoma decipiens*	**JL567**	JN975370	JN975407	[67]
*Cryptosphaeria ligniota*	CBS 273.87	KT425233	KT425168	[68]
*Cryptosphaeria pullmanensis*	ATCC 52655	KT425235	KT425170	[69]
*Cryptosphaeria subcutanea*	CBS 240.87	KT425232	KT425167	[69]
*Cryptovalsa ampelina*	A001	GQ293901	GQ293972	[70]
*Cryptovalsa ampelina*	DRO101	GQ293902	GQ293982	[70]
*Diatrypasimilis australiensis*	**ATCC MYA-3540**	FJ430590	_	[71]
*Diatrype betulae*	**CFCC52416**	MW632943	MW656391	[43]
*Diatrype bullata*	UCDDCh400	DQ006946	DQ007002	[66]
*Diatrype camelliae-japonicae*	GMB0427	OP935172	OP938734	[15]
*Diatrype castaneicola*	**CFCC 52425**	MW632941	MW656389	[43]
*Diatrype disciformis*	CBS 205.87	AJ302437	_	[68]
*Diatrype larissae*	**FCATAS 2723**	OM040384	OM240964	[72]
*Diatrype quercicola*	**CFCC52418**	MW632938	MW656386	[43]
*Diatrype rubi*	**GMB0429**	OP935182	OP938740	[15]
*Diatrype spilomea*	D17C	AJ302433	_	[68]
*Diatrype stigma*	DCASH200	GQ293947	GQ294003	[70]
*Diatrype undulata*	CBS 271.87	AJ302436	_	[68]
*Diatrypella atlantica*	HUEFS 136873	KM396614	KR259647	[2]
*Diatrypella betulae*	**CFCC 52406**	MW632931	MW656379	[43]
*Diatrypella betulicola*	**CFCC 52411**	MW632935	MW656383	[43]
*Diatrypella banksiae*	**CPC 29118**	KY173402	_	[73]
*Diatrypella delonicis*	MFLUCC 15-1014	MH812994	MH847790	[18]
*Diatrypella elaeidis*	**MFLUCC 15-0279**	MN308417	MN340300	[12]
*Diatrypella fatsiae-japonica*	**GMB0422**	OP935184	OP938744	[15]
*Diatrypella favacea*	Isolate 380	KU320616	_	[2]
*Diatrypella favacea*	DL26C	AJ302440	_	Unpublished
*Diatrypella frostii*	UFMGCB 1917	HQ377280	_	[74]
*Diatrypella guiyangensis*	**GMB0414**	OP935188	OP938742	[15]
*Diatrypella heveae*	MFLUCC 17-0368	MF959501	MG334557	[5]
*Diatrypella heveae*	MFLUCC 15-0274	MN308418	MN340301	[12]
***Diatrypella heveae***	**MFLUCC 23-0180**	**OR563997**	**OR591485**	**This study**
*Diatrypella hubeiensis*	**CFCC 52413**	MW632937	_	[43]
*Diatrypella iranensis*	**KDQ18**	KM245033	KY352429	[28]
*Diatrypella longiasca*	**KUMCC 20-0021**	MW036141	MW239658	[21]
*Diatrypella macrospora*	**KDQ15**	KR605648	KY352430	[17]
*Diatrypella major*	Isolate 1058	KU320613	_	[2]
*Diatrypella major*	Strain 7	OP060703	_	Unpublished
***Diatrypella major***	**MFLUCC 23-0177**	**OR564001**	**OR572100**	**This study**
*Diatrypella oregonensis*	DPL200	GQ293940	GQ293999	[70]
*Diatrypella pseudooregonensis*	**GMB:0039**	MW797115	MW81488	[35]
*Diatrypella pulvinata*	H048	FR715523	FR715495	[2]
*Diatrypella tectonae*	**MFLUCC 12-0172a**	KY283084	_	[19]
*Diatrypella verruciformis*	UCROK1467	JX144793	JX174093	[75]
*Diatrypella vulgaris*	HVFRA02	HQ692591	HQ692503	[27]
*Diatrypella yunnanensis*	**VT01**	MN653008	MN887112	[43]
*Eutypa astroidea*	CBS 292.87	AJ302458	DQ006966	[66]
*Eutypa lejoplaca*	CBS 248.87	DQ006922	DQ006974	[66]
*Eutypa leptoplaca*	CBS 287.87	DQ006924	DQ006961	[66]
*Eutypa maura*	CBS 219.87	DQ006926	DQ006967	[66]
*Eutypa sparsa*	3802-3b	AY684220	AY684201	[27]
*Eutypella cerviculata*	**M68**	JF340269	_	[76]
*Eutypella quercina*	**IRANC 2543C**	KX828139	KY352449	[7]
*Eutypella semicircularis*	MP4669	JQ517314	_	[17]
*Halocryptovalsa salicorniae*	**MFLUCC 15-0185**	MH304410	MH370274	[9]
*Halodiatrype avicenniae*	MFLUCC 15-0953	KX573916	KX573931	[3]
*Halodiatrype salinicola*	**MFLUCC 15-1277**	KX573915	KX573932	[3]
*Kretzschmaria deusta*	CBS 826.72	KU683767	KU684190	[77]
***Melanostictus chiangraiensis***	**MFLUCC 23-0178**	**OR571763**	**OR577309**	**This study**
*Melanostictus longiostiolatus*	**MFLU 19-2146**	MW240636	MW775595	[13]
*Melanostictus thailandicus*	**MFLU 19-2123**	MW240630	MW775590	[13]
***Melanostictus thailandicus***	**MFLUCC 23-0179**	**OR564002**	**OR771024**	**This study**
*Monosporascus cannonballus*	**CMM3646**	JX971617	_	Unpublished
*Monosporascus cannonballus*	**ATCC 26931**	FJ430598	_	Unpublished
*Neoeutypella baoshanensis*	**HMAS 255436**	MH822887	MH822888	[18]
*Paraeutypella citricola*	HVVIT07	HQ692579	HQ692512	[27]
*Paraeutypella citricola*	HVGRF01	HQ692589	HQ692521	[27]
*Paraeutypella citricola*	STEU_8182	MF359635	MF359670	[78]
***Paraeutypella citricola***	**MFLUCC 23-0176**	**OR563996**	**OR591489**	**This study**
*Paraeutypella guizhouensis*	**KUMCC 20-0016**	MW039349	MW239660	[21]
*Paraeutypella pseudoguizhouensis*	**GMB0420**	OP935186	OP938748	[15]
*Paraeutypella pseudoguizhouensis*	GMB0421	OP935187	OP938749	[15]
*Paraeutypella vitis*	UCD2291AR	HQ288224	HQ288303	[79]
*Paraeutypella vitis*	UCD2428TX	FJ790851	GU294726	[80]
*Pedumispora rhizophorae*	**BCC44877**	KJ888853	_	[81]
*Pedumispora rhizophorae*	BCC44878	KJ888854	_	[81]
*Peroneutypa curvispora*	HUEFS 136877	KM396641	_	[2]
*Peroneutypa diminutiasca*	**MFLUCC 17-2144**	MG873479	_	[6]
*Peroneutypa indica*	**NFCCI 4393**	MN061368	MN431498	[8]
*Peroneutypa kochiana*	EL53M	AJ302462	_	[1]
*Peroneutypa mangrovei*	**PUFD526**	MG844286	MH094409	[20]
*Peroneutypa polysporae*	**NFCCI 4392**	MN061367	MN431497	[8]
*Pseudodiatrype hainanensis*	**GMB0054**	MW797111	MW814883	[35]
*Pseudodiatrype hainanensis*	GMB0055	MW797112	MW814884	[35]
*Quaternaria quaternata*	CBS 278.87	AJ302469	_	[68]
*Quaternaria quaternata*	GNF13	KR605645	_	[17]
*Vasilyeva cinnamomi*	**GMB0418**	OP935174	OP938737	[15]
*Vasilyeva cinnamomi*	GMB0419	OP935175	OP938738	[15]
*Xylaria hypoxylon*	CBS 122620	AM993141	KX271279	[82]

Ex-type strains are demonstrated in bold; “–” denotes that the sequence is unavailable; sequences generated in the current study are shown in bold.

## 3. Results

### 3.1. Phylogenetic Analysis 

**Figure 1:**
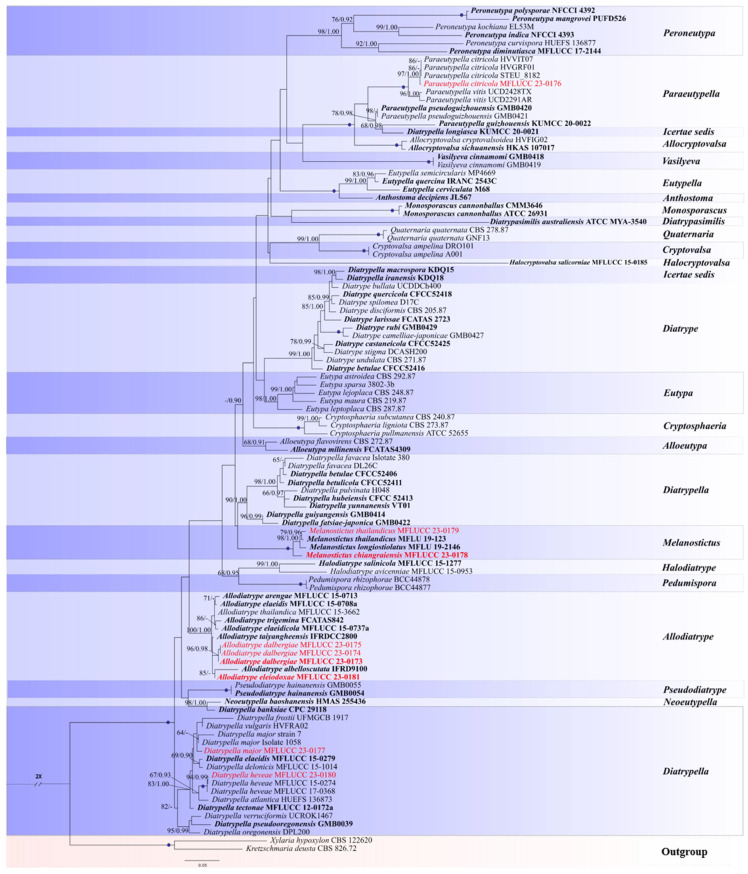
Phylogram generated from maximum-likelihood phylogram analyses of selected taxa in *Diatrypaceae* family based on ITS and *tub*2 matrix. Branch supports of maximum-likelihood (ML) values and Bayesian posterior probability values (BPP) are indicated at the nodes (ML ≥ 60%, left/ BPP ≥ 0.90, right); the tree is rooted with *Kretzschmaria deusta* (CBS 826.72) and *Xylaria hypoxylon* (CBS 122620). Branches with 100% ML/1.00 BPP are shown with a blue dot. Ex-type strains are in black bold. Taxa originating from this study are demonstrated in red.

The phylogenetic trees based on ML and BI analyses of combined DNA sequence (ITS and *tub*2) indicated that the overall topology of the two trees did not have significant differences. Therefore, a tree from the ML method was chosen to represent the evolutionary history of the *Diatrypaceae* family. The dataset for the ingroups comprised 93 strains from 22 genera representing *Diatrypaceae. Xylaria hypoxylon* (L.) Grev. (CBS 122620) and *Kretzschmaria deusta* (Hoffm.) P.M.D. Martin (CBS 826.72) were used as the outgroup taxa (Table 2). The alignment comprised 1549 characters (ITS: 1–602 and *tub*2: 603–1549). The resulting ML tree had a final ML optimization likelihood value of −20181.820962 and is depicted in Figure 1. Parameters for the GTR+F+G4 model of the combined ITS, and *tub*2 were as follows: base frequencies—A = 0.225444, C = 0.273304, G = 0.234138, T = 0.267114; rate parameters—AC = 1.029595, AG = 3.305839, AT = 1.302108, CG = 0.974988, CT = 4.155639, GT = 1.000000; gamma distribution shape alpha—α = 0.393250. The Bayesian posterior probabilities of phylogeny using Markov chain Monte Carlo (MCMC) were assessed with a final average standard deviation of the split frequencies of 0.009926. The phylogenetic tree constructed from the combined ITS and *tub*2 DNA matrix introduces three new species and four host associations within *Diatrypaceae* (Figure 1).

*Diatrypella major* (MFLUCC 23-0177) clustered with *D. major* (Isolate 1058 and Strain 7) with low support. *Diatrypella heveae* (MFLUCC 23-0180) clustered with *D. heveae* (MFLUCC 15-0274) with ML = 94%, BPP = 0.99 support. *Allodiatrype dalbergiae* (MFLUCC 23-0173, MFLUCC 23-0174, MFLUCC 23-0175) and *A. eleiodoxae* (MFLUCC 23-0181) clustered with *Allodiatrype* species. *Allodiatrype dalbergiae* (MFLUCC 23-0173) grouped with *A. albelloscutata* (IFRD9100) and *A. eleiodoxae* (MFLUCC 23-0181). *Allodiatrype dalbergiae* (MFLUCC 23-0173, MFLUCC 23-0174, MFLUCC 23-0175) clustered with ML = 96%, BPP = 0.98 support. *Allodiatrype eleiodoxae* (MFLUCC 23-0181) formed a sister clade with *A. albelloscutata* (IFRD9100) with 85% bootstrap support. *Melanostictus thailandicus* (MFLUCC 23-0179) clustered with the ex-type strain of *M. thailandicus* (MFLU 19-2123) with ML = 79%, BPP = 0.96 supports. Subsequently, *M. chiangraiensis* (MFLUCC 23-0178) formed a distinct clade with other *Melanostictus* species with high statistical support (ML = 100%, BPP = 1.00). *Paraeutypella citricola* (MFLUCC 23-0176), clustered with *P. citricola* strains (HVVIT07, HVVIT01, and STEU _8182) with ML = 97%, BPP = 1.00 bootstrap support.

### 3.2. Taxonomy

#### 3.2.1. *Allodiatrype dalbergiae* N. Afshari and S. Lumyong, sp. nov. (Figure 2)

Index Fungorum number: IF901034; Faces of fungi number: FoF14765. 

Etymology: Epithet refers to the host genus “*Dalbergia*”.

Holotype: MFLU 23-0349. 

Description: Saprobic on *Dalbergia cana* (*Fabaceae*) woody litter. Sexual morph: *Stromata* 0.82–2 × 0.93–2.7 mm ($\bar{x}$ = 1.6 × 1.5 mm, *n* = 10), black, gregarious, erumpent, arising through the cracks in substrate surface, interior well-developed, irregular shaped, multi-loculate. *Stromaticous layer* comprise outer layer of black–olivaceous, firmly packed, and an inner layer of grey, loosely packed parenchymatous cells. *Ostiole* appearing as black spots on the surface of the stromata. *Ascomata* (excluding necks) 250–505(–600) × 125–257 μm ($\bar{x}$ = 393 × 210 μm, *n* = 10), perithecial, immersed and compacted in the stromatic tissue, dark brown–brown, irregular or mostly subglobose, narrowing towards the apex, with separate short neck ostioles. *Ostiolar canal* is cylindrical, periphysate. *Peridium* 17–41 μm wide ($\bar{x}$ = 25 μm, *n* = 40), composed of two layers, outer layer consisting of brown, tightly packed cells, arranged in *textura angularis*, inner layer comprising subhyaline–hyaline, 2–3 thick-walled cells of *textura angularis*. *Paraphyses* composed of 2–6 μm wide ($\bar{x}$ = 3.8 μm, *n* = 60), hyaline, unbranched, filiform, septate, longer than asci. *Asci* spore-bearing section (excluding stalk), (19–)22–34(−40) × 6–10 μm ($\bar{x}$ = 28 × 8 μm, *n* = 35), eight-spored, unitunicate, cylindrical–clavate, apically flat, with J-apical ring, swollen at upper, apex-bearing section (1.9–)2.6–7(−8) μm long ($\bar{x}$ = 4 μm, *n* = 35), long and narrow stalks, stalk-bearing section (16–)22–63(–70) μm long ($\bar{x}$ = 37 μm, *n* = 35). *Ascospores* (6–)7.5–10.5(−11.8) × 1.9–3.7(−4.1) μm ($\bar{x}$ = 9 × 2.5 μm, *n* = 50), overlapping or biseriate, hyaline–pale brown, unicellular, smooth-walled, ellipsoidal–cylindrical or elongate–allantoid, 0–2 guttulate at both ends. Asexual morph: Not observed.

Culture characters: Ascospores germinated on PDA within 24 h, and germ tubes were produced from both end cells. Colonies on PDA, reaching 3 cm diam. after one week at room temperature (25–28 ℃). Colony flat or slightly effuse, dense, irregular, thinner towards the periphery, at upper surface white at the beginning, becoming buff with age, from reverse pale brown at first to dark brown after one month. Pigmentation produced on PDA medium with age.

Material examined: Thailand, Doi Tung National Park, Chiang Rai, on dead wood of *Dalbergia cana*, 26 March 2022, N. Afshari, 4C1T1R1 (MFLU 23-0349, holotype); ex-type living culture MFLUCC 23-0173; on dead wood of *Nayariophyton zizyphifolium*, 6 June 2022, N. Afshari, 1C2T1R3 (MFLU 23-0350, paratype), living culture MFLUCC 23-0174; on dead wood of *Afzelia xylocarpa*, 27 September 2022, N. Afshari, 5C3T2R1 (MFLU 23-0351, paratype), living culture MFLUCC 23-0175.

GenBank accession numbers: MFLUCC 23-0173: ITS = OR571759, *tub*2 = OR771026; MFLUCC 23-0174: ITS = OR571760, *tub*2 = OR591487; MFLUCC 23-0175: ITS = OR571762, *tub*2 = OR771025.

Notes: The combined gene phylogenetic analyses indicated that *A. dalbergiae* (MFLUCC 23-0173) formed a sister clade with *A. albelloscutata* (IFRD9100) and *A. dalbergiae* (MFLUCC 23-0173). *Allodiatrype dalbergiae* (MFLUCC 23-0173) clustered with *A. dalbergiae* (MFLUCC 23-0174) and *A. dalbergiae* (MFLUCC 23-0175) with 96% ML/0.98 BPP support values (Figure 1). Our strain is morphologically distinct from *A. albelloscutata* (IFRD9100) in the size of stroma, ascomata, and peridium and also having subglobous ascomata and a large number of ascomata immersed in a single stroma; however, they almost conform in asci and ascospore size (Table 3) [63]. There are 9/430 bp (2.09%) differences in the ITS sequences of *A. dalbergiae* and *A. albelloscutata* (IFRD9100). Konta et al. [12] considered 1.77–2.14% differences in ITS to introduce a species in this genus. As the *tub*2 sequence is not available for *A. albelloscutata* (IFRD9100) we considered ITS nucleotides coupled with morphological differences to introduce this species [12].

#### 3.2.2. *Allodiatrype eleiodoxae* N. Afshari and S. Lumyong, sp. nov. (Figure 3)

Index Fungorum number: IF901105; Faces of fungi number: FoF14766. 

Etymology: Epithet refers to the host genus “*Eleiodoxa*”.

Holotype: MFLU 23-0357.

Description: Saprobic on *Eleiodoxa* sp. (*Arecaceae*) woody litter. Sexual morph: *Stromata* 1.1–0.8 × 1–2.7 mm ($\bar{x}$ = 0.9 × 0.77 mm, *n* = 10), well-developed interior, superficial, scattered or rarely gregarious on host, comprising black outer layer with smooth or tightly packed, thin parenchymatous cell layer and greenish yellow inner layer with loosely packed parenchymatous cells, with umbilicate ostioles opening to surface of stroma as black spots. *Ascomata* (excluding necks) 195–450 × 170–300(–405) μm ($\bar{x}$ = 288 × 329 μm, *n* = 10), perithecial with groups of 2–5 perithecia immersed in a single stroma, globose–subglobose, black–dark brown, with ostiol. *Ostiolar necks* 100–150 × 50–120 μm ($\bar{x}$ = 140 × 110 μm, *n* = 10), emerging separately, immersed in stromata’s outer layer, cylindrical, sulcate, periphysate. *Peridium* 17–25 μm wide ($\bar{x}$ = 21 μm, *n* = 30), composed of two sections, outer section comprising dark brown, tightly packed cells, arranged in *textura angularis*, inner layer comprising hyaline cells of *textura angularis*. *Hamathecium* comprising 3.5–6 μm wide ($\bar{x}$ = 4.8 μm, *n* = 20) septate, constricted at the septa, wider and flat at the apex, guttulate paraphyses. *Asci* 65–118 × 5.7–9 μm ($\bar{x}$ = 92 × 7.5 μm, *n* = 25), eight-spored, unitunicate, clavate, with long, thin-walled pedicel, upper portion wide, flattened in apex, with J-apical apparatus. *Ascospores* 7–10 × 2.2–3.3 μm ($\bar{x}$ = 9 × 2.8 μm, *n* = 30), unicellular, overlapping, hyaline–pale yellow, allantoid–cylindrical or elongate–allantoid, with small, 2–3 guttulate at both ends, smooth-walled. Asexual morph: Not observed. 

Culture characters: Ascospores germinated on PDA within 24 h, and germ tubes were produced from both end cells. Colonies on PDA, reaching 5 cm diam. after one week at room temperature (25–28 °C). Colony flat, effuse in the center, dense radially fimbriate towards the periphery, from upper surface white to grey, from reverse dark brown or brown at centre becoming radiantly pale brown to the edge. Yellowish brown pigmentation produced on PDA medium at maturity.

Material examined: Thailand, Narathiwat Province, Yi-ngo District, peat swamp forest, on dead wood of *Eleiodoxa* sp., 6 April 2022, O. Karimi, 71-Y (MFLU 23-0357, holotype); ex-type living culture MFLUCC 23-0181.

GenBank accession numbers: ITS: OR571761, *tub*2: OR591484. 

Notes: Based on the phylogram generated from ITS/*tub*2 sequence data, *A. eleiodoxae* (MFLUCC 23-0181) clustered with *A. albelloscutata* (IFRD9100) (85% ML). They have 10/554 bp (1.8%) ITS nucleotide differences. There is a significant difference between the branch length in the phylogenetic tree (Figure 1) and the single ITS gene tree. *Allodiatrype eleiodoxae* (MFLU 23-0357) differs from *A. albelloscutata* (IFRD9100) in larger stromata with 2–5 ascomata, whereas IFRD9100 has 5–11 ascomata [12]. Also, the asci and peridium dimension is considerably larger [12]. However, these two species have no significant differences in the size and shape of ascospores (Table 3). Our species was isolated on *Eleiodoxa* sp. from a peat swamp forest in southern Thailand, whereas *A. albelloscutata* (IFRD9100) was from an unidentified host in a terrestrial habitat in China [63].

#### 3.2.3. *Paraeutypella citricola* (Speg.) L.S. Dissan., Wijayaw., J.C. Kang and K.D. Hyde, Biodivers. Data J. 9: e63864, 14 (2021) [21] (Figure 4)

Index Fungorum number: IF558003; Faces of fungi number: FoF09150.

Synonym: *Eutypella citricola* Speg., in Anales del Museo Nacional de Buenos Aires 6: 245, (1898).

Description: Saprobic on *Microcos paniculata* (*Malvaceae*) woody litter. Sexual morph: *Stromata* immersed to semi-immersed in substrate bark, well-developed interior, carbonaceous, scattered black area on bark and clustered into big groups, circular–irregular. *Ascomata* (excluding necks), 369–570 × 254–540 μm ($\bar{x}$ = 560 × 375 μm, *n* = 10), perithecial, groups of 2–6 perithecia placed in a valsoid arrangement, black or dark brown, globose–subglobose, enclosed with white powdery endostroma, immersed in stromata tissue with ostiole neck. *Ostiolar canal* 240–375 × 122–177 μm ($\bar{x}$ = 290 × 155 μm, *n* = 5), sulcate, papillate, cylindrical, appearing through the bark, with paraphyses in central ostiole canal. *Peridium* 28.5–42 μm wide ($\bar{x}$ = 34 μm, *n* = 40), composed of two layers, hyaline, loosely packed cells of *textura angularis* in the inner layer, dark brown–black compact cells of *textura angularis* in the outer layer. *Hamathecium* composed of 2–7 μm wide ($\bar{x}$ = 4 μm, *n* = 40), hyaline, long, widen at the base, septate, moderately constricted at the septa, unbranched, guttulate, narrowing and apically truncate paraphyses. *Asci* (excluding stalks) 51–76(–87) × 5–9(−9.8) μm ($\bar{x}$ = 59 × 7 μm, *n* = 30), unitunicate, eight-spored, cylindrical–clavate, straight–flexuous, thin-walled, J-apical ring, long pedicel (25–45 μm). *Ascospores* 5–10 × 2–4.4 μm ($\bar{x}$ = 7.5 × 3.4 μm, *n* = 60), overlapping or biseriate, allantoid–sub-allantoid, straight when young and curved at maturity, subhyaline–pale brown, smooth-walled, unicellular, usually guttulate. Asexual morph: Not observed. 

Culture characteristics: Colonies on PDA, reached up to 7 cm diam. after one week at room temperature (25–28 °C), a germ tube was produced from one side. Colony cottony surface, medium dense, slightly effuse, circular and moderately fimbriate towards the periphery, colony from upper surface white to buff, from reverse yellow–pale brown at the centre to cream at the margin. Abundant dots of melanized mycelium expand in the media and are visible from the reverse after one month of incubation on PDA.

Material examined: Thailand, Doi Tung National Park, Chiang Rai, on dead wood of *Microcos paniculata*, 6 June 2022, N. Afshari, 3C2T3R3 (MFLU 23-0352); living culture MFLUCC 23-0176. 

GenBank accession numbers: ITS: OR563996, *tub*2: OR591489.

Notes: Our isolate (MFLU 23-0352) morphologically resembles *P. citricola* (HVVIT07, holotype) by having immersed or semi-immersed stromata, dimension of asci and ascospore, as well as the colour of ascospores [27]. Also, our isolate and *P. citricola* (HMAS 290660) share similar morphological characteristics, for instance, immersed and carbonaceous stromata, sulcate necks, size of ascomata, peridium and paraphysis, and asci ascospores [21]. *Paraeutypella citricola* strains have been reported from *Citrus limon*, *C. paradisi*, *C. sinensis*, *Schinus molle*, *Salix* sp., *Ulmus procera, Vitis vinifera,* and *Acer* sp. [17,21,27]. *Paraeutypella citricola* (MFLU 23-0352) was isolated on *M. paniculata* from Chiang Rai province, Thailand. Based on phylogenetic analysis of combined gene (ITS, *tub*2), MFLUCC 23-0176 clustered with three other *P. citricola* strains (HVVIT07, HVGRF01, STEU 8182) with 97% ML/1.00 BPP statistical supports (Figure 1).

#### 3.2.4. *Diatrypella heveae* Senwanna, Phookamsak and K.D. Hyde, Mycosphere 8 (10): 1846 (2017) [5] (Figure 5) 

Index Fungorum number: IF553859; Faces of fungi number: FoF03775. 

Description: Saprobic on *Microcos paniculata* (*Malvaceae*) woody litter. Sexual morph: *Stromata* 1.2–0.86 × 1–0.7 mm ($\bar{x}$ = 1 × 0.86 mm, *n* = 10), well-developed interior, black, circular–irregular in shape, coriaceous, solitary to rarely 2–4 gregarious, scattered, erumpent, in vertical margins bark of substrate adhering to stromata, white powdery–yellowish pigment entostroma, with brown loosely packed pseudoparenchymatous cells around the entostroma. *Ascomata* (495–)420–253 × (210–)290–340 μm ($\bar{x}$ = 355 × 290 μm, *n* = 10), perithecial, with groups of 3–10 perithecia, black, globose–subglobose, immersed in stromata. *Ostiolar canal* 100–190 μm × 65–90(–114) μm ($\bar{x}$ = 147 × 84 μm, *n* = 8), sulcate, filled with paraphyses. *Peridium* 26–42 μm wide ($\bar{x}$ = 34 μm, *n* = 30), composed of two layers of *textura angularis*; cells of inner layer hyaline, cells of outer layer dense and brown. *Hamathecium* composed of 2–5 μm wide ($\bar{x}$ = 4 μm, *n* = 40), hyaline, septate, unbranched, guttulate, apically truncate paraphyses. *Asci* (including stalks) 138–90 × 11–19 μm ($\bar{x}$ = 113.5 × 15 μm, *n* = 20), unitunicate, polysporous, thin-walled, clavate, rounded at the apex, J-apical ring. *Ascospores* 5–8 × 1.3–2.2 μm ($\bar{x}$ = 6.6 × 1.7 μm, *n* = 60), overlapping, cylindrical to allantoid or elongate–allantoid, subhyaline to light brown, smooth-walled, aseptate, at maturity straight and guttulate at both ends. Asexual morph: Not observed. 

Culture characteristics: Colonies on PDA reached up to 7.5–8 cm diam. after one week at room temperature (25–28 °C), a germ tube emerges from one end cell. Colony flat or effuse, irregular, diffuse in the margin, from above white, from reverse radiating from pale brown to pale yellow outwardly.

Material examined: Thailand, Doi Tung National Park, Chiang Rai, on dead wood of *Microcos paniculata*, 27 September 2022, N. Afshari, 3C3T3R1 (MFLU 23-0354); living culture MFLUCC 23-0180.

GenBank accession numbers: ITS: OR563997, *tub*2: OR591485.

Notes: *Diatrypella heveae* (MFLU 23-0354) is morphologically similar to *D. heveae* (MFLUCC 17-0368) in size, shape and colour of asci and ascospores and erumpent ascomata with bark adhering to host epidermis, but differs in having smaller stroma, ascomata and the ostiolar canal [5]. All three strains of *D. heveae* (MFLUCC 17-0368, MFLUCC 15-0274, and MFLUCC 23-0180) were isolated from Chiang Rai, Thailand but from different hosts [5,12]. According to our phylogenetic analyses based on ITS and *tub*2 sequence data (Figure 1), *D. heveae* (MFLUCC 23-0180) clustered with *D. heveae* (MFLUCC 17-0368 and MFLUCC 15-0274) with strong statistical support (100% ML/1.00 BPP). Based on phylogenetic results and morphological overlap, our strain is introduced as *D. heveae.*

#### 3.2.5. *Diatrypella major* (Berl.) Lar.N. Vassiljeva, Fungal Diversity 19: 198 (2005) [83] (Figure 6) 

Synonym: *Diatrypella decorata* var. major Berl., Icon. Fung. (Abellini) 3(3-4): 119 (1902)

Index Fungorum number: IF344628; Faces of fungi number: FoF14767. 

Description: Saprobic on *Nayariophyton zizyphifolium* (*Malvaceae*) woody litter. Sexual morph: *Stromata* 0.6–1.2 × (0.5–)0.8–1 mm ($\bar{x}$ = 0.9 × 77 μm, *n* = 10), single or gregarious, scattered on the substrate, semi-immersed, surrounded by bark’s epidermis, black, pustulate, rounded to irregular in shape, with 2–8 ascomata, endostromata comprises inner layer of white, loose, parenchymal cells and outer layer of dark brown–black, small, dense, thin parenchymal cells. *Ascomata* (excluding neck) (210–)300–488 × 193–400 μm ($\bar{x}$ = 303 × 390 μm, *n* = 10), perithecial, globous–mostly subglobous, immersed in stroma, black, with cylindrical neck. *Ostiole canal* 700–750 μm high, 300–450 μm diam. ($\bar{x}$ = 300 × 350 μm, *n* = 10), sulcate, centric, opening separately, ring-like furrow absent, periphysate. *Peridium* 14.5–27 μm wide ($\bar{x}$ = 20 μm, *n* = 30), composed of cells arranged in *textura angularis* and thin-walled cells, outer layer brown, inner layer hyaline cells. *Hamathecium* composed of 2.2–4.4 μm wide ($\bar{x}$ = 3.4 μm, *n* = 30), hyaline, aseptate, unbranched, guttulate, apically truncate paraphyses. *Asci* 101.5–142(−150) × 14–19 μm ($\bar{x}$ = 113 × 16.5 μm, *n* = 20), polysporous, clavate, moderately short-stalked, apically rounded, J-apical ring. *Ascospores* 5–9(−11) × 1.7–3 μm ($\bar{x}$ = 7.8 × 2.4 μm, *n* = 70), overlapping, allantoid, slightly curved, aseptate, smooth-walled, hyaline or pale yellow–yellowish in mass. Asexual morph: Not observed.

Culture characteristics: Ascospores germinating on PDA within 24 h. Colonies on PDA reaching 4.5 cm diam. After one week at room temperature (25–28 °C). Colony medium dense, fimbriate towards the edge, from above white at the beginning, became pale brown in centre with age, from reverse pale brown at the centre and yellow towards the margin.

Material examined: Thailand, Doi Tung National Park, Chiang Rai, on dead wood of *Nayariophyton zizyphifolium*, 26 March 2022, N. Afshari, 1C1T3R5a (MFLU 23-0353), living culture MFLUCC 23-0177.

GenBank accession numbers: ITS: OR564001, *tub*2: OR572100.

Note: The comparison of the ITS sequences revealed that *D. major* (MFLUCC 23-0177) is 99% similar to *D. major* (Isolate 1058). Based on the phylogenetic analysis in Figure 1, *D. major* (MFLUCC 23-0177) formed a separate lineage; however, it clustered with *D. major* (Isolate 1058 and Strain 7) without bootstrap support. *Diatrypella major* (Isolate 1058 and Strain 7) only has ITS sequence data. Therefore, the combined analysis of ITS and *tub*2 sequence data could not clearly define the position of *D. major* (MFLUCC 23-0177). Vasilyeva and Stephenson [83] provided a short description for *D. major*; it has small stromata with sulcate ostioles similar to our strain but smaller asci and ascospores. As there was not enough morphological data to compare in detail, we considered the ITS sequence similarity between the *D. major* (MFLUCC 23-0177) and the two strains of *D. major. Diatrypella major* has been reported in the United States [2,83]. This study provides additional data for *D. major* from the dead wood of *N. zizyphifolium* in Thailand for the first time.

#### 3.2.6. *Melanostictus chiangraiensis* N. Afshari and S. Lumyong, sp. nov (Figure 7)

Index Fungorum number: IF901106; Faces of fungi number: FoF14768. 

Etymology: Epithet refers to the province “Chiang Rai” where the holotype was collected.

Holotype: MFLU 23-0355.

Description: Saprobic on *Dalbergia cana* woody litter (*Fabaceae*). Sexual morph: *Ascomata* 195–290 × 170–378 μm ($\bar{x}$ = 226 × 253 μm, *n* = 10), immersed, raised areas visible as black dots in the host tissue, solitary or aggregated, scattered, mostly distributed evenly, globose or mostly subglobous, ectostroma yellow. *Ostiole canal* 80–40 × 48–27 μm ($\bar{x}$ = 57 × 36 μm, *n* = 5), short, central, sulcate at top, periphysate. *Peridium* 25–42 μm ($\bar{x}$ = 34 μm, *n* = 30) wide, coriaceous, 2-layered, outer layer comprising brown–dark brown, thick, dense cells of *textura angularis*, inner layer comprising hyaline, big cells of *textura angularis. Paraphyses* 2–7 μm ($\bar{x}$ = 5 μm, *n* = 40) wide, septate, constricted at septa, unbranched, guttulate, longer than asci, narrow towards tip, with a blunt end. *Asci* (36–)40–53(–58) × 4–6 μm ($\bar{x}$ = 47 × 5 μm, *n* = 25), eight-spored, unitunicate, clavate, thin-walled, pedicel 12–23 μm ($\bar{x}$ = 18 μm, *n* = 20), moderately long, developing from the base of the ascomata, apically truncate, apical ring J- and minute. *Ascospores* 4–7(–7.5) × 1–1.7 μm ($\bar{x}$ = 5.5 × 1.3 μm, *n* = 50), L/W 4.2, hyaline–pale yellow, overlapping, aseptate, smooth-walled, elongate–allantoid, slightly curved. Asexual morph: Not observed.

Culture characteristics: Colonies on PDA, reaching 5 cm diam. after one week at room temperature (25–28 °C). Colony circular to slightly irregular, narrower at margin, flat, leather surface, a colony from the front, and reverse buff.

Material examined: Thailand, Doi Tung National Park, Chiang Rai, on dead wood of *Dalbergia cana*, 7 July 2022, N. Afshari, 4C2T2R2 (MFLU 23-0355, holotype); ex-type living culture MFLUCC 23-0178. 

GenBank accession numbers: ITS: OR571763, *tub*2: OR577309.

Notes: In the combined gene phylogeny, *M. chiangraiensis* (MFLUCC 23-0178) formed a separate and distinct clade within *Melanostictus* (100% ML/1.00 BPP) (Figure 1). The ITS base pair comparisons of *M. chiangraiensis* with *M. longiostiolatus* and *M. thailandicus* revealed 16/540 (3%) and 19/540 (3.5%) bp differences (excluding gaps) and 22/643 (3.4%) and 14/637 (2.2%) bp differences (excluding gaps) between the *tub*2 sequences. Samarakoon et al. [13] observed J^+^ apical ring in the asci of *M. thailandicus* and *M. longiostiolatus,* but we only observed the asci with J^-^ apical ring. Besides, *M. thailandicus* and *M. longiostiolatus* have bigger ascomata, ostiolar canal and asci [13]. The peridium size and shape of *M. chiangraiensis* conform with *M. thailandicus,* while the paraphyses are similar to *M. longiostiolatus* (Table 4). We isolated *M. chiangraiensis* (MFLU 23-0355) from *D. cana* woody litter, but the other two species were isolated from unidentified hosts from northern Thailand [13].

#### 3.2.7. *Melanostictus thailandicus* Samarak. and K.D. Hyde, Fungal Diversity 112: 35 (2022) [13] (Figure 8)

Index Fungorum number: IF558721; Faces of fungi number: FoF10198 

Description: Saprobic on *Dalbergia cultrata* (*Fabaceae*) woody litter. Sexual morph: *Ascomata* (excluding neck) 250–500 × 247–575 μm ($\bar{x}$ = 313 × 363 μm, *n* = 10), solitary or gregarious, scattered, immersed in the substrate and slightly raised to surface and visible as black dots on the bark, globose or mostly subglobose, rarely flattened at the base, ectostroma grey to white. *Ostiole canal* (105–)133–311 × 81–125 μm ($\bar{x}$ = 192 × 104 μm, *n* = 5), central, sulcate, periphysate. *Peridium* 22–40 μm ($\bar{x}$ = 33.5 μm, *n* = 30) wide, 2-layered, outer layer comprising brown cells of *textura angularis*, inner layer composed of hyaline cells of *textura angularis*. *Paraphyses* 2.7–5.5 μm ($\bar{x}$ = 4 μm, *n* = 30) long, guttulate, septate, unbranched, slightly constricted at septa, truncate at end. *Asci* (38.5–)44–68.5 × 3.6–5.7 μm ($\bar{x}$ = 55 × 4.6 μm, *n* = 30), eight-spored, clavate, unitunicate, thin-walled, apically truncate. *Ascospores* (4.1–)4.6–6.3 × 1–1.9 μm ($\bar{x}$ = 5.3 × 1.4 μm, *n* = 50), L/W 3.8, overlapping or biseriate, hyaline, cylindrical or elongate–allantoid, aseptate, smooth-walled, guttulate at both ends. Asexual morph: Not observed.

Culture characteristics: Ascospores germinate on PDA within 24 h, reaching up to 4.5 cm diam. After one week at room temperature (25–28 °C). Colony on PDA, flat, narrow towards the edge, from front white at first became yellow–pale brown, reverse white to pale yellow at the margin, buff at the centre.

Material examined: Thailand, Doi Tung National Park, Chiang Rai, on dead wood of *Dalbergia cultrata*, 7 July 2022, N. Afshari, 6C2T1R2 (MFLU 23-0356), living culture MFLUCC 23-0179. 

GenBank accession numbers: ITS: OR564002, *tub*2: OR771024.

Notes: We isolated and illustrated *M. thailandicus* (MFLUCC 23-0179) from Chiang Rai, Thailand, associated with *D. cultrata* woody litter. The morphological characteristics largely resembled those of *M. thailandicus* (MFLU 19-2123) (e.g., the size of ascomata, ostiolar canals, peridium, paraphysis, asci, and ascospores) (Table 4) [13]. In the phylogenetic analyses, *M. thailandicus* (MFLUCC 23-0179) is sister to *M. thailandicus* (MFLU 19-2123) with 79% ML/0.96 BPP support values (Figure 1). The sequence data of ITS and *tub*2 are similar to those of the ex-type.

**Table 3:** The morphology of *Allodiatrype* species.

*Allodiatrype* Species	Ascostromata	Ascomata	Ostiolar Canals	Peridium	Paraphyses	Asci	Ascospores	Hosts	Countries	References
*A. albelloscutata* (IFRD9100)	680–820 × 910–1560 μm, well-developed interior, 5–11 ascomata	230–270 × 300–380 μm, immersed, globose–subglobose	154 × 30 μm, cylindrical, periphysate	5–10 μm	N/A	43–82 × 6–7 μm, swollen and rounded upper portion, J-apical ring, eight-spored, unitunicate	7–11 × 2–3 μm, biserriate or irregular, overlapping, light brown, ovoid to elongate–allantoid	Unidentified plant	China	[63]
*A. arengae* (MFLUCC 15-0713)	690–940 × 370–935 μm, well-developed interior, 1–5 ascomata	(excluding necks) 250–400 × 240– 400 μm, immersed, globose– subglobose	100–170 × 70–130 μm, cylindrical, sulcate periphysate	12–25 μm	3–7 μm, septate, hyaline	spore-bearing part (14–)20−45 × (4–)6–10(−12) μm, apically rounded, J-apical ring, eight-spored, unitunicate	(6–)7–10(−12) × 2–3 μm, overlapping, yellowish–light brown, ellipsoidal–cylindrical or elongate–allantoid	*Arenga pinnata*	Thailand	[12]
*A. elaeidicola* (MFLUCC 15-0737a)	1.2–2.8 × 0.96–1.66 mm, well-developed interior	(excluding necks) 280–430 × 180–435 μm, immersed, globose–subglobose	120–185 × 60–120 μm	14–40 μm	N/A	spore-bearing part (17–)20–31(−43) × 4–7 μm, apically rounded, J-apical ring, eight-spored, unitunicate	(6.5–)8–10(−11) × 1.5–3 μm, overlapping, yellowish–brown, ellipsoidal–cylindrical or elongate–allantoid	*Elaeis guineensis*	Thailand	[12]
*A. elaeidis* (MFLUCC 15-0708a)	470–860 × 440–710 μm, well-developed interior, bi–multi-ascomata	(excluding necks) 250–350 × 230–300 μm, immersed, globose–subglobose	100–130 × 95–115 μm, cylindrical, sulcate, periphysate	20–40 μm	2–7 μm, filiform, longer than asci, septate, branched, hyaline	spore-bearing part (17–)20–30(−39) × 9–11(−14) μm, apically rounded, J-apical ring, eight-spored, unitunicate	(6–)8–10(−11) × 1.5–3) μm, overlapping, yellowish–pale brown, ellipsoidal–cylindrical or elongate–allantoid	*Elaeis guineensis*	Thailand	[12]
***A. eleiodoxae*** **(MFLUCC 23-0181)**	**1–0.8 × 1–2.7 mm, well-developed interior,** **2–5 ascomata**	**(excluding necks) 195–450 × 170–300(–405) μm, immersed,** **globose–subglobose**	**100–150 × 50–120 μm, cylindrical, periphysate**	**17–25 μm**	**3.5–6 μm, septate, constricted at the septa, wider and flat at the apex, guttulate**	**65–118 × 5.7–9 μm including stalk, upper portion wide, flattened in apex, J-apical ring, eight-spored, unitunicate**	**7–10 × 2.2–3.3 μm, overlapping, hyaline–pale yellow, allantoid–cylindrical or elongate–allantoid**	***Eleiodoxa* sp.**	**Thailand**	**This study**
*A. taiyangheensis* (IFRDCC2800)	710–980 × 1430–2290 μm, well-developed interior, 5–15 ascomata	320–530 × 230–300 μm, immersed, globose–subglobose	cylindrical, periphysate	12–17 μm	N/A	spore-bearing part 32–58 × 6–7 μm, flat at apex, eight-spored, unitunicate	7–10 × 2–3 μm, biserriate or irregular, overlapping, yellowish, ellipsoidal–cylindrical or elongate–allantoid	Unidentified plant	China	[63]
*A. thailandica* (MFLUCC 15-3662)	1–1.2 mm wide, erumpent, 4 ascomata	226–336 × 177–235 μm, immersed, globose–subglobose, narrowing towards the apex	periphysate	6.5–15 μm	2.2–4.5 μm, septate, longer than the asci, wider at the apex	55–80 × 5–7μm, narrow, thick-walled, swollen upper portion, apex flat, J-apical ring, eight-spored, unitunicate	3.8–6.9 × 1–1.4 μm, multi-seriate to overlapping pale brown, allantoid–cylindrical	Unidentified plant	Thailand	[64]
*A. trigemina* (FCATAS842)	0.81–3.23 × 0.61–1.72 mm, 5–7 ascomata	308–680 × 157–376 μm, immersed, globose–subglobose	N/A	N/A	hyaline	spore-bearing part 16–43 × 6–12 μm, spores arranged tightly, J+ apical ring, eight-spored, unitunicate	(5.5–)6–8(–9.2) × (1.5–)2.1–2.3(–2.5) μm, ellipsoidal to cylindrical, multi-seriate to overlapping, arranged at the tip of the asci, colorless	Unidentified plant	China	[65]
***A. dalbergiae* (MFLUCC 23-0173)**	**0.82–2 × 0.93–2.7 mm,** **interior well-developed,** **multi-loculate**	**(excluding necks) 250–505(–600) × 125–257 μm, immersed, irregular, or mostly subglobose, narrowing towards the apex**	**cylindrical, periphysate**	**17–41 μm**	**2–6 μm, hyaline, unbranched, filiform, septate, longer than asci**	**spore-bearing part (19–)22–34(−40) × 6–10 μm, apically flat, J-apical ring, swollen at upper, eight-spored, unitunicate**	**(6–)7.5–10.5(−11.8) × 1.9–3.7(−4.1) μm, overlapping or biseriate, hyaline–pale brown, ellipsoidal–cylindrical or elongate–allantoid**	***Dalbergia cana*, *Nayariophyton zizyphifolium*, and *Afzelia xylocarpa***	**Thailand**	**This study**

The symbol “N/A” denotes no data available or not mentioned in reference papers, and the species from this study are indicated in bold.

**Table 4:** The morphology of *Melanostictus* species.

*Melanostictus* Species	Ascomata	Ostiolar Canals	Peridium	Paraphyses	Asci	Ascospores	Hosts	Countries	References
*M. thailandicus* (MFLU 19-2123)	415–580 × 300–410 μm, immersed, slightly raised, solitary or aggregated, clusters or evenly distributed, globose or subglobose, base rarely flattened	185–280 × 85–145 μm, centric, periphysate, sulcate on top	27–40 μm	2.4–4 μm, long, septate, constricted at septa, smooth-walled, ends blunt	50–64 × 3.8–5 μm, eight-spored, unitunicate, clavate, thin-walled, apical ring minute	5–6.2 × 1–1.7 μm, L/W 3.9, overlapping, cylindrical or elongate–allantoid, hyaline, guttulate	Unidentified plant	Thailand	[13]
*M. longiostiolatus* (MFLU 19-2146)	550–630 × 300–370 μm, immersed, slightly raised, visible as black dots, solitary or aggregated, mostly in pairs, clusters or evenly distributed, globose	300–390 × 110–180 μm, centric, periphysate, sulcate on top.	30–38 μm	3–7.5 μm, wider at the base, septate, rarely branched, constricted at septa, smooth-walled, blunt end	50–65 × 5.5–8 μm, clavate, thin-walled, eight-spored, unitunicate, apical ring minute, developing from the base, apically flattened	3.5–5.5 × 1–1.5 μm, L/W 3.75, overlapping, hyaline, cylindrical or elongate–allantoid	Unidentified plant	Thailand	[13]
***M. chiangraiensis*** **(MFLUCC 23-0178)**	**195–290 × 170–378 μm, immersed, solitary or aggregated, scattered, mostly distribute evenly, globose or mostly subglobose**	**80–40 × 48–27 μm, short, central, sulcate at top, periphysate**	**25–42 μm**	**2–7 μm, septate, constricted at septa, unbranched, guttulate, longer than asci, narrow towards tip, blunt end**	**36–)40–53(–58) × 4–6 μm, eight-spored, unitunicate, clavate, thin-walled, developing from the base of the ascomata, apically truncate, apical ring J- and minute**	**4–7(–7.5) × 1–1.7 μm, L/W 4.2, hyaline–pale yellow, overlapping, elongate–allantoid, slightly curved**	***Dalbergia cana***	**Thailand**	**This study**

The species from this study are indicated in bold.

## 4. Discussion

**Figure 2:**
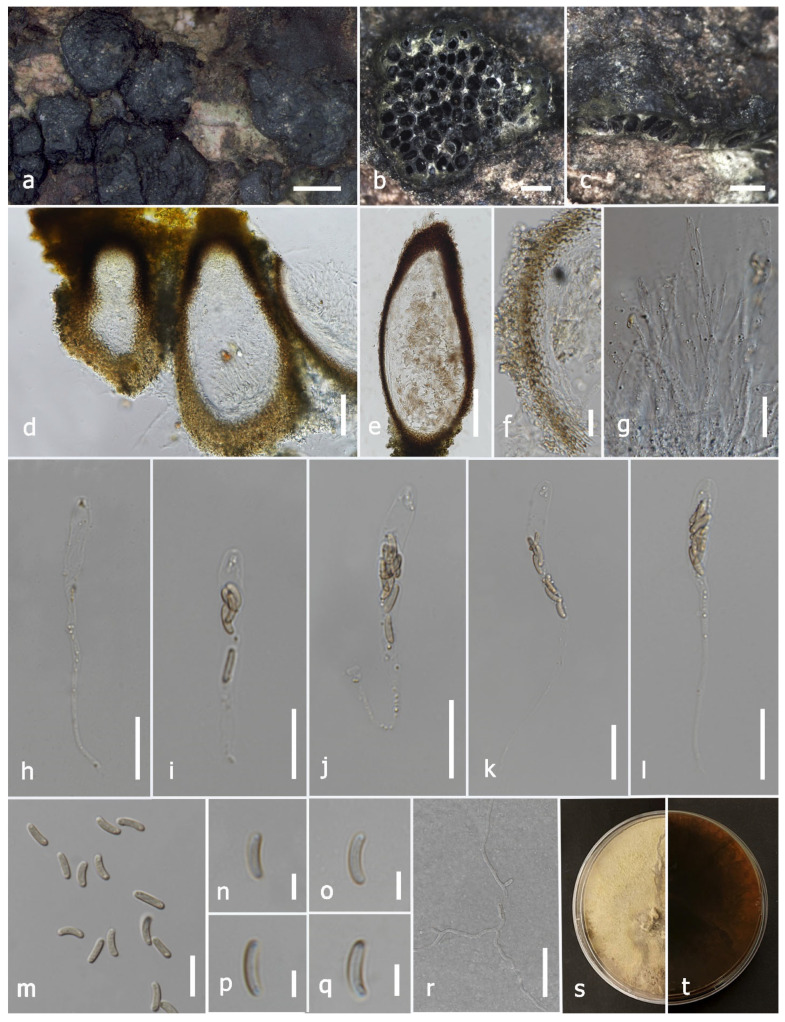
*Allodiatrype dalbergiae* (MFLU 23-0349, holotype). (**a**) Close-up of stromata on *Dalbergia cana* woody litter. (**b**) Transverse section of the stroma. (**c**) Longitudinal section of the stroma. (**d**,**e**) Vertical section through ascoma. (**f**) Section of peridium. (**g**) Paraphyses. (**h**–**l**) Asci. (**m**–**q**) Ascospores. (**r**) A germinated ascospore. (**s**,**t**) Colony on PDA. Scale bars: (**a**) = 1 mm, (**b**,**c**) = 500 μm, (**d**,**e**) = 100 μm, (**f**–**l**) = 20 μm, (**m**,**r**) = 10 μm, (**n**–**q**) = 5 μm.

**Figure 3:**
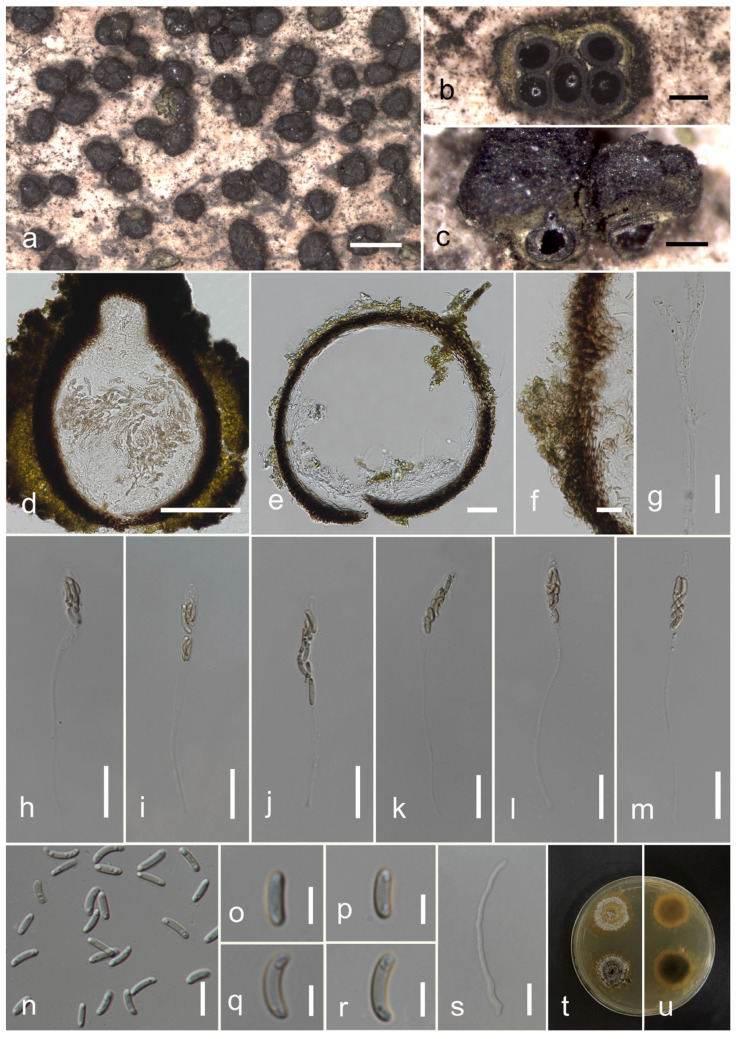
*Allodiatrype eleiodoxae* (MFLU 23-0357, holotype). (**a**) Close-up of stromata on *Eleiodoxa* sp. woody litter. (**b**) Transverse section of stroma. (**c**) Longitudinal section of stroma. (**d**,**e**) Vertical section through ascoma. (**f**) Section of peridium. (**g**) Paraphyses. (**h**–**m**) Asci. (**n**–**r**) Ascospores. (**s**) A germinated ascospore. (**t**,**u**) Colony on PDA. Scale bars: (**a**) = 1 mm, (**b**,**c**) = 200 μm, (**d**) = 100 μm, (**e**) = 50 μm, (**f**–**m**,**s**) = 20 μm, (**n**) = 10 μm, (**o**–**r**) = 10 μm.

**Figure 4:**
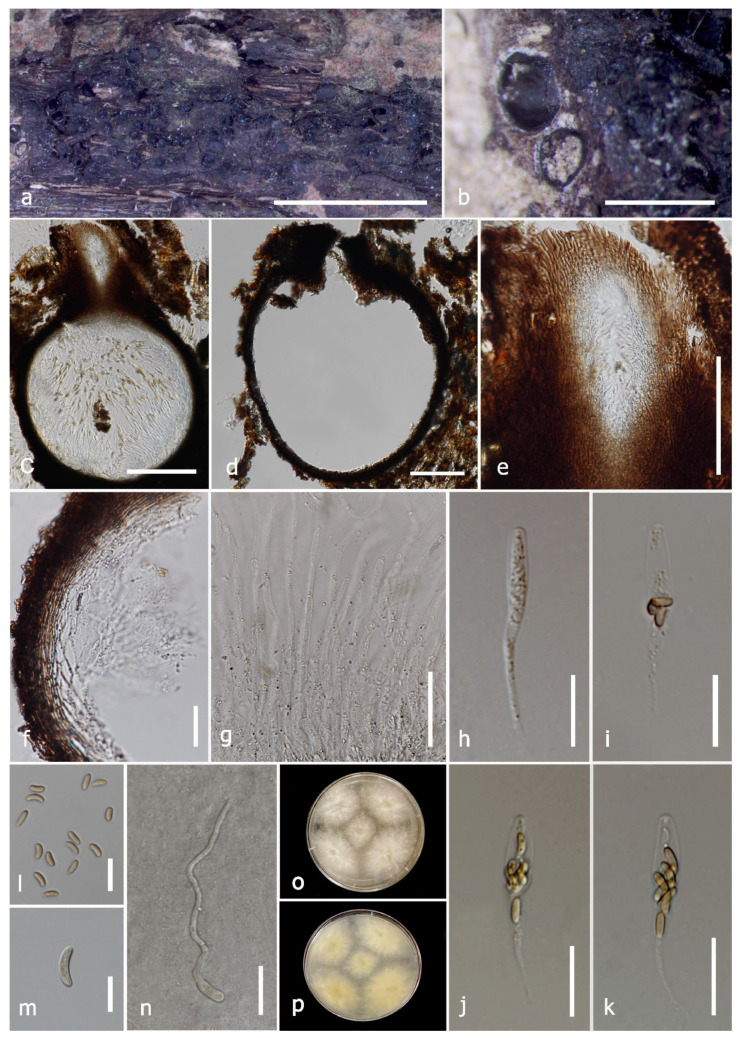
*Paraeutypella citricola* (MFLU 23-0352, new host record). (**a**) Close-up of stromata on *Microcos paniculata* woody litter. (**b**) Longitudinal section of the stroma. (**c**,**d**) Vertical section through ascoma. (**e**) Ostiol canal. (**f**) Section of peridium. (**g**) Paraphyses. (**h**–**k**) Asci. (**l**,**m**) Ascospores. (**n**) A germinated ascospore. (**o**,**p**) Colony on PDA. Scale bars: (**a**) = 1 mm, (**b**,**c**) = 200 μm, (**d**,**e**) = 100 μm, (**g**) = 50 μm, (**f**,**h**–**k**,**n**) = 20 μm, (**l**,**m**) = 5 μm.

**Figure 5:**
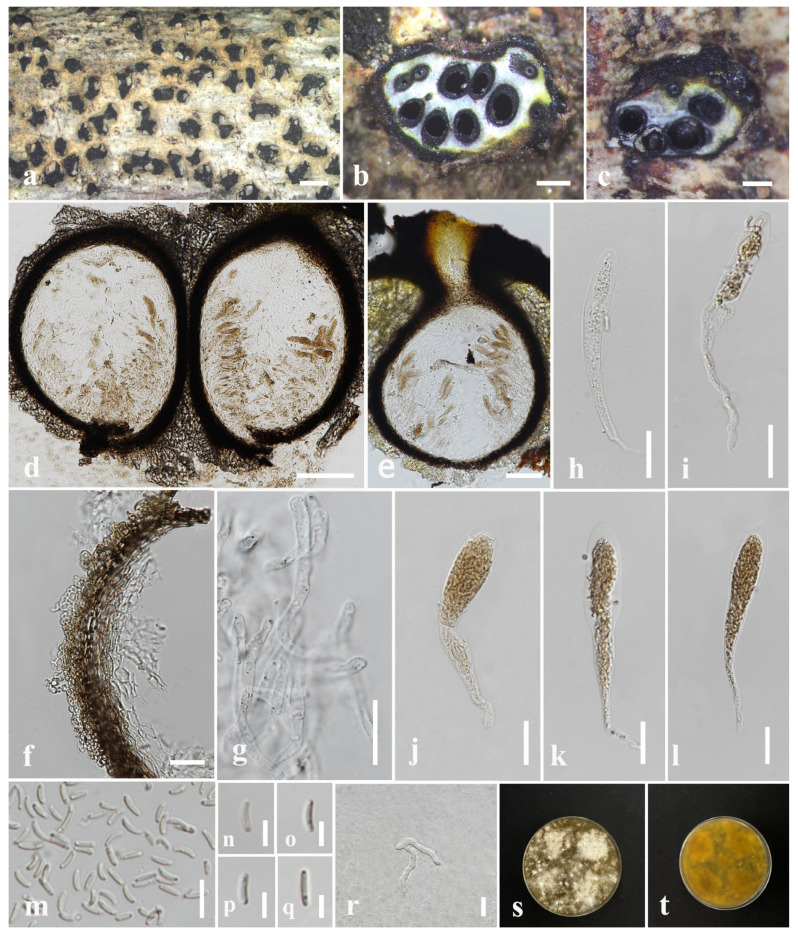
*Diatrypella heveae* (MFLU 23-0354, new host record). (**a**) Close up of stromata on *Microcos paniculata* woody litter. (**b**) Transverse section of the stroma. (**c**) Longitudinal section of the stroma. (**d**,**e**) Vertical section through ascoma. (**f**) Section of peridium. (**g**) Paraphyses. (**h**–**l**) Asci. (**m**–**q**) Ascospores. (**r**) A germinated ascospore. (**s**,**t**) Colony on PDA. Scale bars: (**a**) = 1 mm, (**b**,**c**) = 200 μm, (**d**,**e**) = 100 μm, (**f**–**l**) = 20 μm, (**m**,**r**) = 10 μm, (**n**–**q**) = 5 μm.

**Figure 6:**
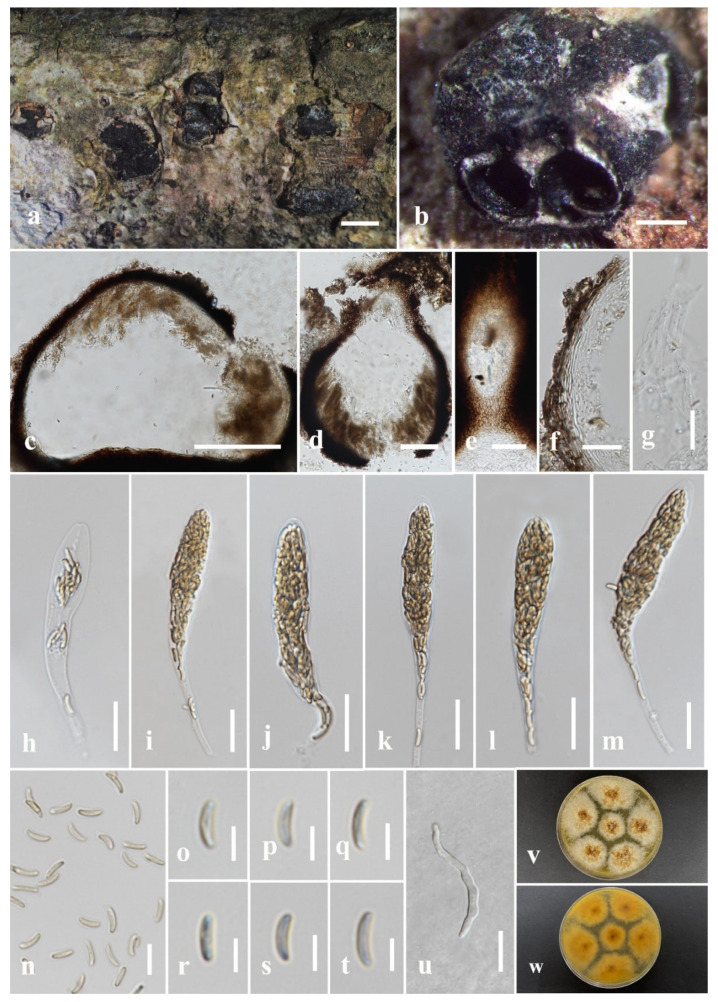
*Diatrypella major* (MFLU 23-0353, new host and geographical record). (**a**) Close-up of stromata on *Nayariophyton zizyphifolium* woody litter. (**b**) Longitudinal section of the stroma. (**c**,**d**) Vertical section through ascoma. (**e**) Ostiol canal. (**f**) Section of peridium. (**g**) Paraphyses. (**h**–**m**) Asci. (**n**–**t**) Ascospores. (**u**) A germinated ascspore. (**v**,**w**) Colony on PDA. Scale bars: (**a**) = 1 mm, (**b**) = 200 μm, (**c**,**d**) = 100 μm, (**e**) = 50 μm, (**f**–**m**) = 20 μm, (**n**,**u**) = 10 μm, (**o**–**t**) = 5 μm.

**Figure 7:**
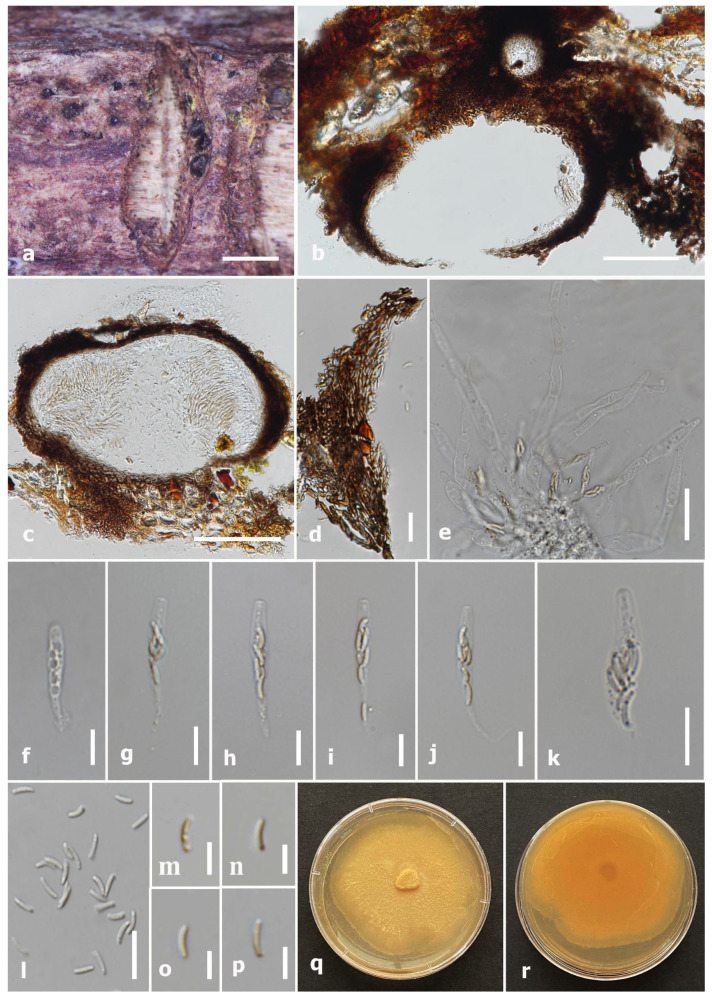
*Melanostictus chiangraiensis* (MFLU 23-0355, holotype). (**a**) Longitudinal section of stroma on *Dalbergia cana* woody litter. (**b**,**c**) Vertical section through the stroma. (**d**) Section of peridium. (**e**) Paraphyses. (**f**–**k**) Asci. (**l**–**p**) Ascospores. (**q**,**r**) Colony on PDA. Scale bars: (**a**) = 500 μm, (**b**,**c**) = 100 μm, (**d**,**e**) = 20 μm, (**f**–**l**) = 10 μm, (**m**–**p**) = 5 μm.

**Figure 8:**
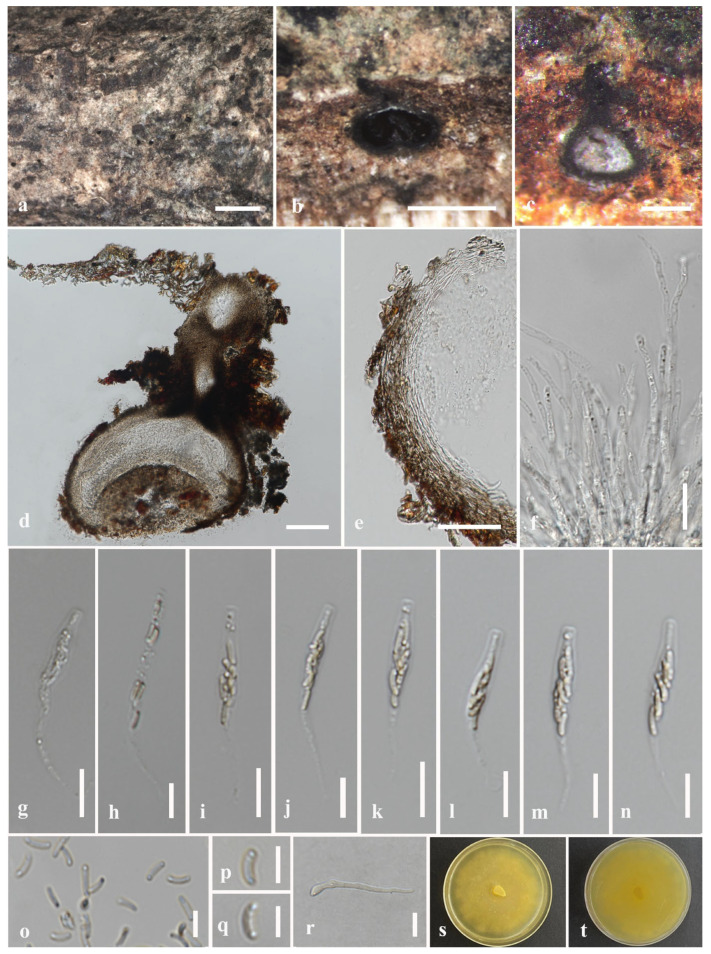
*Melanostictus thailandicus* (MFLU 23-0356, new host record). (**a**) Close-up of stromata on *Dalbergia cultrata* woody litter. (**b**,**c**) Longitudinal section of the stroma. (**d**) Vertical section through stroma. (**e**) Section of peridium. (**f**) Paraphyses. (**g**–**n**) Asci. (**o**–**q**) Ascospores. (**r**) A germinated ascspore. (**s**,**t**) Colony on PDA. Scale bars: (**a**) = 1 mm, (**b**) = 500 μm, (**c**) = 200 μm, (**d**) = 100, (**e**) = 50 μm, (**f**) = 20 μm, (**g**–**n**) = 10 μm. (**o**–**r**) = 5 μm.

In this study, we collected nine isolates of *Diatrypaceae* when studying wood-inhabiting microfungi in terrestrial habitats in northern Thailand and from a peat swamp forest in the southern area of the country. Three new species, one new geographical report, and four new host records were introduced in *Diatrypaceae* based on the polyphasic approach (morpho-molecular, Figure 1, Figure 2, Figure 3, Figure 4, Figure 5, Figure 6, Figure 7 and Figure 8) recommended by [84,85]. Recently, a significant number of new species and genera of diatrypaceous fungi have been described [2,7,12,13,15,21,65]. Nevertheless, *Diatrypaceae* species are difficult to distinguish only by morphological characteristics; however, some features are still substantial to identify them (e.g., size of asci, ascospores, peridium, paraphysis, and apical apparatus) [12,13,51].

The above criteria support the introduction of *A. dalbergiae* and *A. eleiodoxae* (Figure 1, Figure 2 and Figure 3). *Allodiatrype* was erected by Konta et al. [13] to accommodate *A. arengae* (the type species), *A. elaeidicola*, *A. elaeidis*, and *A. thailandica* (syn. *Diatrype thailandica*). This genus resembles *Diatrype* but differs in the number of ascomata (1–10 immersed ascomata), with or without a black stromatic zone. In contrast, the stromata of *Diatrype* primarily spread in a large area [12]. Besides, phylogenetically, these fungi clustered in an entirely separated clade [12], as also evidenced by this study (Figure 1). 

We also introduced *M. chiangraiensis* from *D. cana* woody litter (Figure 1 and Figure 7). *Melanostictus* was introduced in *Diatrypaceae* by Samarakoon et al. [13] to accommodate *M. longiostiolatus* (type species) and *M. thailandicus.* This genus is distinct from *Halodiatrype* and *Pedumispora*; however, all of these genera possess the characteristics of having immersed ascomata, papillate ostioles, and eight-spored, unitunicate asci [3,86]. *Melanostictus* differs from the other two genera in having yellow to white ectostroma, larger papillate ostioles, and aseptate, cylindrical or elongate–allantoid ascospores [13]. Our collection, *M. chiangraiensis*, is phylogenetically and morphologically related to *M. longiostiolatus* and *M. thailandicus* (Figure 1, Figure 7 and Figure 8, Table 4). However, it has a J^-^ apical ring and smaller ascomata, ostiolar canal and asci than *M. longiostiolatus* [13].

The placement of species in some genera of *Diatrypaceae* is confusing as they are paraphyletic or polyphyletic [12,19]. Shang et al. [19] stated that the phylogenetic analyses of *Cryptosphaeria* and *Eutypella* genera do not show concordance with morphology, maybe due to the low number of species representing each genus [19]. This agrees with the phylogenetic results of *Allodiatrype* species. Besides, some genera, like *Melanostictus*, have immersed ascomata [13], which are difficult to notice. Therefore, it is more likely to find novel species with precise observation for future studies, particularly from substrates without dominant and significant fruiting bodies. Additionally, phylogenetic analysis based on ITS cannot properly resolve this family [31]. Therefore, morphological features coupled with phylogeny based on the ITS+*tub*2 sequence dataset are needed to identify the species of *Diatrypaceae* [12,21,51]. However, molecular data for many taxa are unavailable in the GenBank database, confusing phylogenetic analysis, particularly for closely related taxa.

The ITS and *tub*2 sequences, the primary markers used for the phylogeny of *Diatrypaceae*, are insufficient to clarify the taxonomy of all family members. Therefore, introducing some species in *Diatrypaceae* requires a revision with the study of fresh collections and multilocus phylogeny of ITS+LSU+SSU+*tub*2+*rpb*2+*tef*1-α datasets. Using multiple genetic loci for analysis is pivotal. It implies that data obtained from multiple genetic markers provides more comprehensive and accurate insights into the relationships among distinct taxa within the *Diatrypaceae*. Therefore, the lack of genetic information hampers the precise study and classification of some species’ placement within this family, posing a challenge to a comprehensive understanding of their taxonomy. This emphasizes the need for further studies to fill this important gap. In addition, appropriate morphological characters are essential to determine their placement within the family.

Furthermore, since *Diatrypaceae* species are recorded on a range of hosts in different habitats, they do not seem to have a host preference [21]. This is similar to the results of this research; *A. dalbergiae* was found on three different hosts (*D. cana*, *N. zizyphifolium*, and *A. xylocarpa*). In addition, *A. eleiodoxae* species was isolated from palm litter in peat swamp forests, reinforcing that species of *Allodiatrype* can be found in different habitats [12,15,65]. Also, most *Allodiatrye* species are isolated from Thailand and China, whereas all *Melanostictus* are from Thailand, which suggests a rich diversity in these countries [87,88]. We isolated the two introduced species from a protected area (Doi Tung National Park), showing the potential to explore new fungi in conserved environments and regions to expose novel fungal taxa.

One of the significance of research in a protected area is identifying and documenting the fungi diversity important for preserving biodiversity in specific regions. In other words, understanding the diversity of fungi contributes to ecosystem investigations in different aspects. As fungi play crucial roles in the decomposition of dead materials, nutrient cycling, and symbiotic relationships with plants, discovering the diversity of fungal taxa helps in the functioning of ecosystems. Besides, protected areas like Doi Tung National Park are usually undisturbed, and conducting research in these areas has a high potential for discovering novel and endemic fungal species, consequently contributing to the expansion of fungal diversity.

## Figures and Tables

**Table 1:** Details of genes/loci with primers and PCR conditions.

Genes/Loci	Primers (Forward/Reverse)	Thermal Cycles *	References
ITS	ITS5/ITS4	94 °C: 30 s, 56 °C: 50 s, 72 °C: 60 s	[47]
*tub*2	Bt2a/Bt2b and T1/Bt2b	94 °C: 30 s, 55 °C: 50 s, 72 °C: 90 s	Modified from [48]
	T1/T22	95 °C: 60 s, 54 °C: 110 s, 72 °C: 120 s	Modified from [49]

* Initial denaturation at 94 °C for 3 min, final extension at 72 °C for 10 min, final hold at 4 °C, with 40 cycles for all gene regions.

## Data Availability

All sequences generated in this study were submitted to GenBank [https://www.ncbi.nlm.nih.gov, accessed on 19 September 2023].
